# Endoscopic Resection Compared with Gastrectomy to Treat Early Gastric Cancer: A Systematic Review and Meta-Analysis

**DOI:** 10.1371/journal.pone.0144774

**Published:** 2015-12-10

**Authors:** Shuanhu Wang, Zongbing Zhang, Mulin Liu, Shiqing Li, Congqiao Jiang

**Affiliations:** Department of Gastrointestinal Surgery, The First Affiliated Hospital of Bengbu Medical College, Bengbu, Anhui, China; University Hospital Llandough, UNITED KINGDOM

## Abstract

**Background:**

Endoscopic resection and gastrectomy are treatment modalities for early gastric cancer, but their relative benefits and risks are unclear. We conducted a systematic review and meta-analysis to compare endoscopic resection and gastrectomy for treating early gastric cancer.

**Methods:**

We searched PubMed, Embase, and the Cochrane Library until April 2015 for studies comparing endoscopic resection with gastrectomy for treatment of early gastric cancer. Outcome measures were five-year overall survival (OS), length of hospital stay and postoperative morbidity. We calculated pooled hazard ratio (HR), weighted mean difference (WMD) and odds ratio (OR) using random effects models.

**Results:**

Six studies comprising 1,466 patients (618 endoscopic resection and 848 gastrectomy) met inclusion criteria. Five-year OS was similar between endoscopic resection and gastrectomy (HR, 1.06; 95%CI: 0.61 to 1.83). Endoscopic resection was associated with shorter hospital stays (WMD, -6.94; 95%CI: -7.59 to -6.29) and reduced overall postoperative morbidity (OR, 0.36; 95%CI: 0.17 to 0.74).

**Conclusions:**

While five-year OS is similar between endoscopic resection and gastrectomy, endoscopic resection offers a shorter hospital stay and fewer complications than gastrectomy for treating early gastric cancer. Endoscopic resection is a reasonable treatment for early gastric cancer with a negligible risk of lymph node metastasis.

## Introduction

Gastric cancer is the fifth most common malignancy and the third leading cause of cancer death worldwide [1]. Prognosis is poor mainly due to late stage diagnoses. Compared with advanced gastric cancer, early gastric cancer has an excellent prognosis, and the five-year survival rate exceeds 90% [2, 3]. Early gastric cancer detection is increasing and represents 60% of all gastric cancer cases in Japan [4].

Radical gastrectomy is the traditional treatment for early gastric cancer and can be used not only to remove the primary tumor, but also to remove the lymph node. Early gastric cancer offers excellent long-term outcomes after surgical curative resection [5]. However, radical gastrectomy is associated with considerable morbidity and poor quality of life [6, 7]. A minimally invasive approach could lead to a better outcome. Since the 1980s, endoscopic mucosal resection (EMR) has been used. Endoscopic submucosal dissection (ESD) was developed in the late 1990s. Endoscopic resection including EMR and ESD has been gradually applied to patients with early gastric cancer [8, 9]. So far, endoscopic resection for early gastric cancer is now widely used in many countries and the proportion of endoscopic resections for early gastric cancer is also increasing [10, 11]. Endoscopic treatment of early gastric cancer offers superior quality of life and cheaper cost, but the risks and benefits compared with traditional gastrectomy are unclear [12, 13]. To address this deficit, we conducted a systematic review and meta-analysis to compare endoscopic resection and gastrectomy for treating early gastric cancer.

## Materials and Methods

We used a predefined study protocol that defined search strategy, inclusion criteria, outcome measures, study quality appraisal and statistical methods *a priori*.

### Literature search and inclusion criteria

Two authors (S.W. and Z.Z.) searched electronic databases including Pubmed, Embase, and the Cochrane Library until April 2015. Search terms included “stomach neoplasms”, “stomach cancer”, “endoscopic surgery”, “endoscopic mucosal resection”, “endoscopic submucosal dissection”, “endoscopic resection”, and “gastrectomy”. The search had no language restrictions. In addition we searched references of studies to identify related studies. If duplicated data were presented in several studies, only the most recent or largest study was included.

Studies meeting the following criteria were eligible: (1) population: newly diagnosed early gastric cancer patients who had no previous treatment; (2) intervention: endoscopic resection (EMR or ESD or both) for early gastric cancer met the absolute or expanded indication; (3) comparison: gastrectomy performed with either an open or laparoscopic method; (4) outcome measure: five-year OS; (5) study design: all types of study.

Early gastric cancer is defined as lesions in the mucosa or submucosa, regardless of lymph node metastasis [14]. The absolute indications for endoscopic resection are nonulcerated differentiated intramucosal cancers ≤2 cm in diameter [15]. The expanded indications for endoscopic resection are as follows:1) nonulcerated differentiated intramucosal cancers without limitation of tumor size; 2) ulcerated differentiated intramucosal cancers measuring ≤3 cm; 3) differentiated minute submucosal cancer ≤3 cm (SM1,≤500 μm); and 4) nonulcerated undifferentiated intramucosal cancers ≤3 cm [16].

### Data extraction, outcome measures and quality assessment

Two authors independently assessed all titles and abstracts for relevance and extracted the data (S.W. and S.L.), and disagreements were resolved through discussion. When no consensus could be reached, a third specialist was consulted (C.J.). The primary outcome was five-year OS. Secondary outcomes included hospital stay and overall postoperative morbidity. Five-year OS was used to estimate treatment efficacy while hospital stay and overall postoperative morbidity were used to estimate perioperative risks. Overall postoperative morbidity were described as early and late or described as treatment related morbidity and systemic morbidity. Morbidities within 30 days were defined as early morbidities, and those occurring beyond 30 days were defined as late morbidities. Study quality was assessed using the Newcastle-Ottawa quality assessment scale (range, 0 to 9 stars) by two independent authors (S.W. and M.L.) [17].

### Statistical analysis

Weighted mean difference (WMD) with 95% confidence interval (CI) was used to analyze continuous outcomes. When a study reported a median instead of mean, and a range or interquartile range instead of standard deviation, the mean and standard deviation were estimated according to methods described in the Cochrane handbook or the method described by Hozo et al [18]. OS was evaluated with pooled HR and their 95% CI. None of the included articles directly reported HR and 95% CI, so statistical methods are used to calculate them [19]. GetData Graph Digitizer was used to read Kaplan-Meier curves for included studies and an HR calculation spreadsheet was used to estimate HR and 95% CI. For postoperative morbidity we estimated pooled OR and 95% CI.

Statistical heterogeneity was assessed with *I* ^*2*^ and χ^2^statistics. Heterogeneity was considered significant if the *P* value (χ^2^) was <0.1 and *I* ^*2*^was >50%. A random effects model was used even if no significant statistical heterogeneity was noted. This takes into account the low statistical power of tests of heterogeneity and the likelihood that clinical heterogeneity may exist even if statistical heterogeneity cannot be demonstrated [20]. Whenever significant heterogeneity was present, we performed subgroup analyses to explore the potential sources of heterogeneity. These subgroups were based on two factors—the type of intervention (EMR vs. ESD) and region of patients (Asia vs. beyond Asia). Sensitivity analysis was conducted by omitting one study at a time to assess the influence of each single study on the overall risk estimate. Because of the limited number (below 10) of studies included in each analysis, publication bias was not assessed [21].

Statistical analysis was performed with Review Manager (RevMan, version 5.3. The Nordic Cochrane Centre, the Cochrane Collaboration. Copenhagen, Denmark).

## Results

### Search results

**Fig 1:**
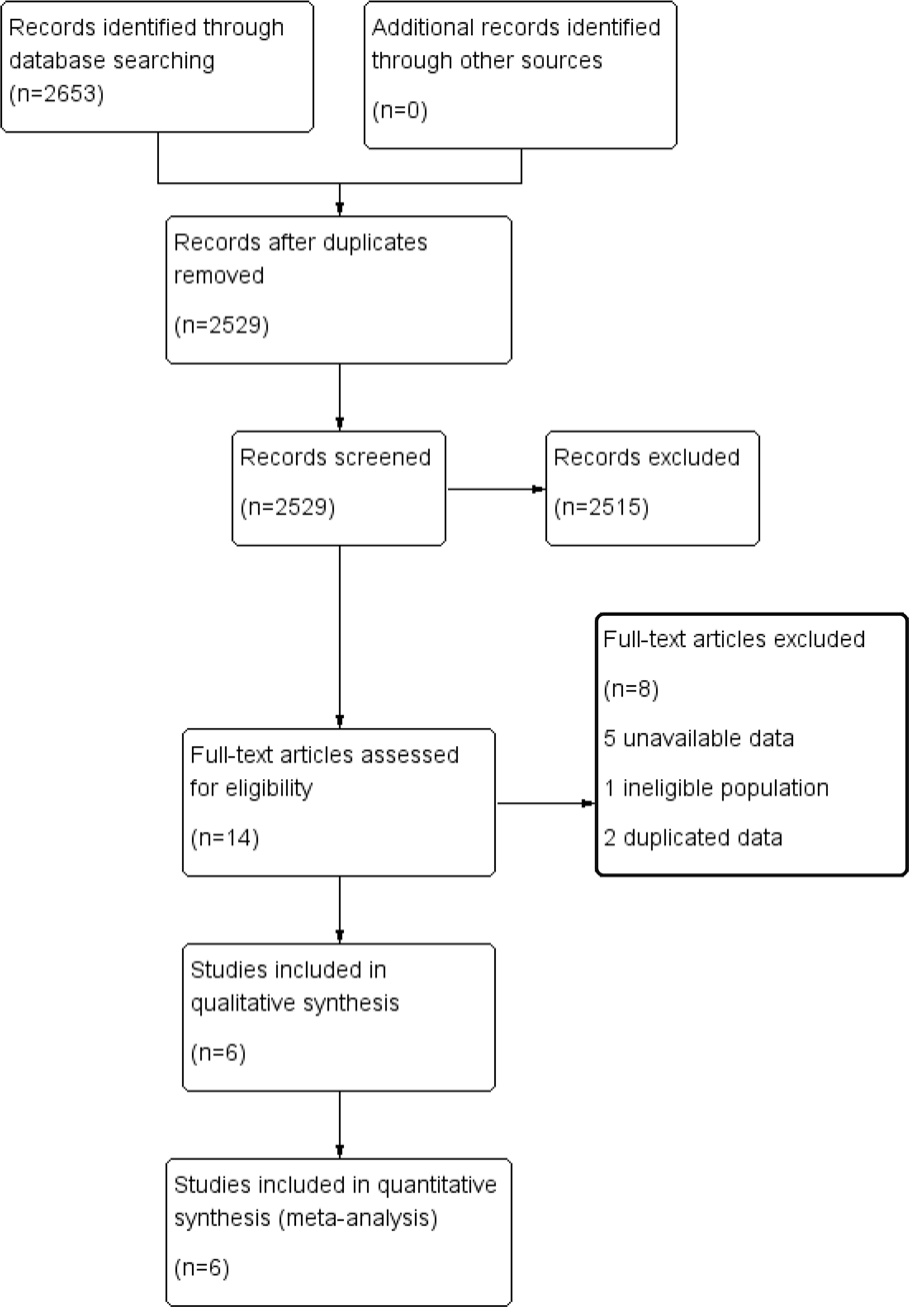
Flow chart of study screening and selection.

We initially identified 2,653 articles and we removed 124 duplicated articles. After reading titles and abstracts, 2,515 studies were excluded for being irrelevant, leaving 14 for full-text review. Upon further review, 8 articles were excluded for the following reasons: five articles with unavailable data [22–26], one article with ineligible population and two articles with duplicated data [27–29]. Finally, six articles were included [30–35]. (Fig 1).

### Study characteristics

Included studies were published between 2005 and 2015. Six studies comprising 1,466 patients were included in the meta-analysis. This included 618 patients undergoing endoscopic resection and 848 patients receiving gastrectomy. Study sample size ranged from 38 to 551 patients. Five studies were published by Asian scholars, and one article was written by North America authors, likely due to the higher incidence of gastric cancer in Asian countries, especially East Asia. A summary of study characteristics is presented in Table 1 and Table 2, and quality assessment is shown in Table 3.

**Table 1:** Details of the articles included.

				Sample size		
Reference	Year	Country	Journal	ER	gastrectomy	Type of study
Chiu et al [30]	2012	Hong Kong, China	Surg Endosc	74	40	retrospective cohort study
Kim et al [31]	2014	Korea	Gut Liver	142	71	cohort study
Etoh et al [32]	2005	Japan	Gastrointest Endosc	49	44	retrospective cohort study
Choi et al [33]	2011	Korea	Gastrointest Endosc	172	379	retrospective cohort study
Kim et al [34]	2015	Korea	Endoscopy	165	292	retrospective cohort study
Najmeh et al [35]	2014	Canada	Gastroenterology	16	22	cohort study

ER, endoscopic resection.

**Table 2:** Characteristics of the included articles.

	Approach	Ageyr	Comorbid disease N (%)	Follow-up duration, months	Size of tumor cm	Histologic type N (%)		Depth of invasion N (%)		Location of tumor N (%)		
Reference				median	mean±SD,	differentiated	undifferentiated	mucosa	submucosa	upper	middle	lower
Chiu et al [30]	ESD	66.3±12.3	18(24.3)	27.0	1.9±0.5			66(89.2)	8(10.8)			
	gastrectomy	67.0±12.8	25(62.5)	77.6	2.5±0.8			19(47.5)	21(52.5)			
Kim et al [31]	ESD	62.0±10.3		76.7		127(89.4)	15(10.6)	135(95.1)	7(4.9)	9(6.3)	65(45.8)	68(47.9)
	gastrectomy	56.7±12.0		65.5		54(76.1)	17(23.9)	62(87.3)	9(12.7)	8(11.3)	1(1.4)	62(87.3)
Etoh et al [32]	EMR	84.2	34(69.4)	57	1.3±0.6	44(89.8)	5(10.2)	39(79.6)	10(20.4)			
	gastrectomy	82.2	25(56.8)	57	2.8±0.5	40(90.9)	4(9.1)	15(34.1)	29(65.9)			
Choi et al [33]	EMR	59.3±9.1	69(40.1)	81	1.6±1.0	156(90.7)	16(9.3)	172(100)	0(0)	11(6.4)	41(23.8)	120(69.8)
	gastrectomy	58.4±10.3	126(33.3)	88	1.7±1.1	342(90.2)	37(9.8)	379(100)	0(0)	25(6.6)	93(24.5)	261(68.9)
Kim et al [34]	ER	62.0±17.8	62(37.5)	49.2	2.5±0.9	156(94.5)	9(5.5)	130(78.8)	35(21.2)	10(6.1)	24(14.5)	131(79.4)
	gastrectomy	60.0±11.9	135(46.1)	59.3	3.0±1.2	261(89.4)	31(10.6)	250(85.6)	42(14.4)	21(7.2)	42(14.4)	229(78.4)
Najmeh et al [35]	ESD	68.3±11.3			2.0±0.7							
	gastrectomy	72.0±8.8			3.8±2.1							

ER, endoscopic resection

ESD, endoscopic submucosal dissection

EMR, endoscopic mucosal resection

SD, standard deviation

Differentiated type denotes well-differentiated or moderately differentiated adenocarcinoma Undifferentiated type denotes poorly differentiated adenocarcinoma or signet ring-cell carcinoma.

**Table 3:** Quality assessment of included articles.

	Selection	Comparability	Outcome	Total
Reference	(0–4)	(0–2)	(0–3)	(0–9)
Chiu et al [30]	****	*	**	7
Kim et al [31]	****	*	**	7
Etoh et al [32]	***	*	***	7
Choi et al [33]	****	**	**	8
Kim et al [34]	****	*	**	7
Najmeh et al [35]	****	*	*	6

### Five-year OS

**Fig 2:**
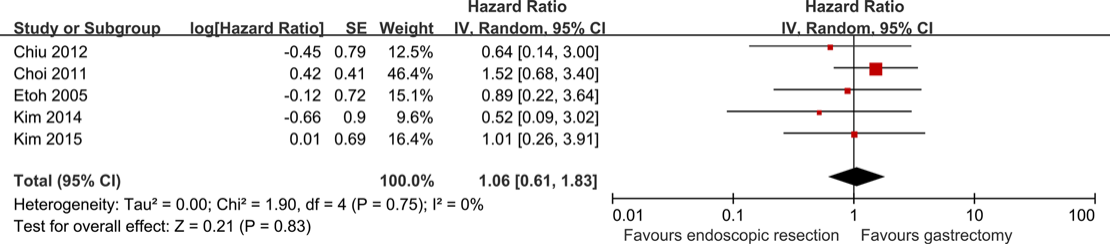
Forest plot of five-year OS.

All studies but one provided OS curves [35]. No paper directly provided HR and 95% CI for OS. We calculated HR and 95% CI for five manuscripts [30–34] with statistical methods but we could not calculate HR and 95% CI for one paper due to missing data [35]. Pooling data revealed that five-year OS for endoscopic resection was similar with that for gastrectomy (HR, 1.06; 95% CI: 0.61 to 1.83; *P* = 0.83) (Fig 2).

### Hospital stay

**Fig 3:**
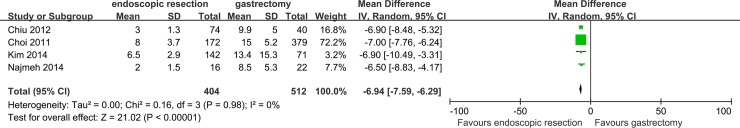
Forest plot of hospital stay.

Four studies reported hospital stay and there was no significant heterogeneity among the studies (*P* = 0.98, *I* ^*2*^ = 0%). In the random effects model, hospital stay was shorter by nearly 7 days in the endoscopic resection group (WMD, -6.94; 95%CI: -7.59 to -6.29; *P* <0.00001) (Fig 3).

### Overall postoperative morbidity

**Fig 4:**
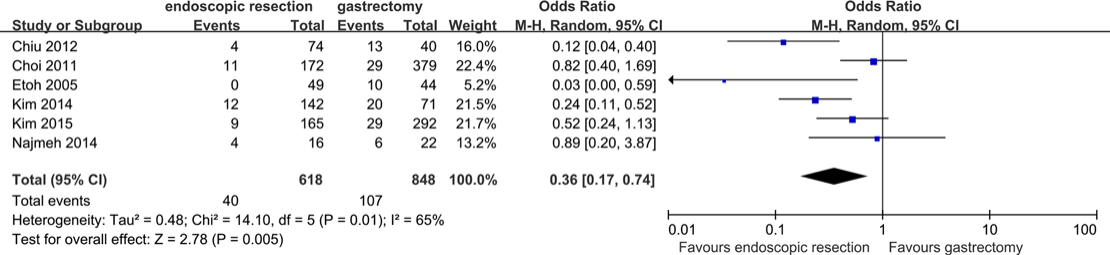
Forest plot of overall postoperative morbidity.

All studies reported overall postoperative morbidity and studies were significantly heterogeneous (*P* = 0.01, *I* ^*2*^ = 65%). In the random effects model, the overall postoperative morbidity of patients undergoing endoscopic resection was less than for those undergoing gastrectomy (OR, 0.36; 95%CI: 0.17 to 0.74; *P* = 0.005) (Fig 4). Subsequently, subgroup analyses were conducted to explore the potential source of heterogeneity. Similar results were observed in subgroup analyses, with substantial evidence of heterogeneity (Table 4).

**Table 4:** Subgroup analyses by the type of intervention and region of patients.

Analysis	Studies,N	ER, N	Gastrectomy,N	OR, 95%CI	*P* value for association	*P* value for heterogeneity	*I* ^*2*^
Type of intervention:							
EMR	2	221	423	0.22(0.01, 5.97)	0.90	0.02	81%
ESD	3	232	133	0.27(0.10, 0.69)	0.007	0.11	54%
Region of patients:							
Asia	5	602	826	0.31(0.14, 0.69)	0.004	0.01	69%
Beyond Asia	1	16	22	0.89(0.20, 3.87)	NA	NA	NA

ER, endoscopic resection;

NA, not available.

### Sensitivity analysis

We performed sensitivity analysis excluding study at a time. The pooled HR for the five-year OS remained similar between the two groups, ranging from 0.78 (95% CI: 0.37 to 1.64, *P* = 0.51) to 1.14 (95% CI: 0.64 to 2.05, *P* = 0.66). Length of hospital stay did not materially change ranging from -6.98 (95% CI: -7.65 to -6.30, *P*<0.00001) to -6.94 (95% CI: -7.60 to -6.28, *P*<0.00001). Similarly the effect on overall postoperative morbidity did not significantly change (pooled OR ranged from 0.28 [95% CI: 0.13 to 0.62, *P* = 0.002] to 0.45 [95% CI: 0.22 to 0.91, *P* = 0.03]).

## Discussion

The aim of this study was to compare perioperative and oncological outcomes between endoscopic resection and gastrectomy for treating early gastric cancer. The results from our meta-analysis revealed that, compared with gastrectomy, endoscopic resection shortened hospital stay, reduced overall postoperative morbidity and made no significant difference in terms of five-year OS.

These results are similar to those published in a previous meta-analysis by Bennett et al [36]. However, the type of intervention was different in the two studies. The previous analysis only included EMR. With the progress of technology, ESD has been widely used for treating early gastric cancer. Compared with EMR for early gastric cancer, ESD showed considerable advantages regarding en bloc resection rate and histologically complete resection rate [37]. So the present study included not only EMR but also ESD. The previous study, on the other hand, assessed survival at the five-year survival rates. Our meta-analysis used methods of survival analysis and expressed the treatment effect as a HR. That was considered a more powerful tool than assessment of survival at five years only.

We found no significant differences in five-year OS between each group, Lymph node metastasis is the most important prognostic factor for early gastric cancer [38]. Zheng’s group [39] reported that the five- and ten-year survival rates were significantly lower in patients with lymph node metastases which are low-frequency events (2.6–4.6% of mucosal cancers) in early gastric cancer with mucosal invasion [16, 40]. However, the incidence of lymph node metastasis in submucosal cancers has been reported as approximately 20% [41, 42]. The submucosa is divided into 3 layers: SM1, SM2, and SM3 according to their depth and some reports indicate that these metastases began in the SM3 layer [43–45]. The possibility of lymph node metastasis is almost zero in early gastric cancer with a size less than 30mm, well differentiated histology, and submucosal penetration of less than 500μm [16]. Most tumors were confined to the mucosa in the endoscopic resection group, and submucosal invasion occurred in the SM1 layer only. Thus, with appropriate case selection, endoscopic resection can offer similar long-term survival compared to gastrectomy.

We documented a distinct advantage for endoscopic resection over gastrectomy with respect to hospital stay, which was shorter, and overall postoperative morbidity which was less (6.5%) than for those undergoing gastrectomy (12.6%).Early and late complications in the endoscopic resection group included bleeding and perforation, which were commonly reported in the literature [46, 47], at rates of 4.3% and 5.3%, respectively [48]. Almost all bleeding and perforation were successfully managed by endoscopic procedures as previously reported. There were other complications in the gastrectomy group, such as wound infection, intestinal obstruction, wound dehiscence, anastomosis stricture, respiratory disease [49]. Late and systemic complications occurred in the surgery group only. Radical gastrectomy with lymph node dissection was performed by an open or laparoscopic approach and most articles reported no differences in complication rates [50, 51]. Some complications of gastrectomy required endoscopic treatment, others required surgical interventions.

The indications for endoscopic resection were based on the zero risk for lymph node metastasis obtained from a large number of surgical cases. Though many clinical and pathological features of the lesions were assessed to determine which factors predicted the presence of nodal metastases, Prediction was sometimes not entirely accurate. The lymph node positive rate for the surgical resection group was reported as 10% and 13.6% in two included articles [30, 32], respectively. The remaining articles did not report the positive rate of lymph nodes. With the development of methods for identifying the risk of lymph node metastasis, we believe that patients will be more accurately selected.

We followed clear methodology, such as predefined inclusion criteria, outcome measures, study quality appraisal and statistical methods *a priori*. The majority of the studies we included were high quality studies awarded seven or more stars. We conducted sensitivity analysis to assess the influence of individual studies on the result and sensitivity analysis indicated that the results were robust. However, our study also has limitations including the lack of randomized controlled trials (RCT), and inclusion of a relatively few number of studies. Second, clinical and pathological characteristics between endoscopic resection and gastrectomy groups were inconsistent. There were more mucosal tumors, more differentiated tumors and smaller tumor sizes in the endoscopic resection group. These factors may have positively biased outcomes against the endoscopic resection group. In contrast, a lower rate of *en bloc* resection may have negatively biased outcomes against the endoscopic resection group. Third, heterogeneity existed in the meta-analysis for overall postoperative morbidity. Although subgroup analyses were performed, we did not detect the major source of heterogeneity. Finally, the patient sample size for all included studies was relatively small indicating low statistical power.

In conclusion, endoscopic resection is associated with similar five-year OS, shorter hospital stays, reduced overall postoperative morbidity compared with gastrectomy for the treatment of early gastric cancer. This suggests that endoscopic resection is a reasonable alternative to gastrectomy for the treatment of early gastric cancer with a negligible risk of lymph node metastasis. However, because these findings are based on observational studies with potential for bias and confounding, a well-powered, multicenter, randomized, controlled trial is needed to confirm these findings.

## Supporting Information

S1 File PRISMA checklist. (DOC)

S2 File Search strategy in Embase. (DOC)

S3 File PLOS ONE Clinical Studies Checklist. (DOCX)
