# Supplementary material for: Endoscopic Resection Compared with Gastrectomy to Treat Early Gastric Cancer: A Systematic Review and Meta-Analysis
Source: PLoS One. 2015 Dec 10;10(12):e0144774. doi: 10.1371/journal.pone.0144774 (PMC4686077; doi:10.1371/journal.pone.0144774)
Supplement: S2 File — (DOC) [file pone.0144774.s002.doc]

Search strategy in Embase

#1 'stomach cancer'/exp

#2 'cancer, stomach':ab,ti

#3 'gastric cancer':ab,ti

#4 #1 OR #2 OR #3

#5 'gastrectomy'/exp

#6 'gastric resection':ab,ti

#7 'gastroresection':ab,ti

#8 'hemigastrectomy':ab,ti

#9 'resection, gastric':ab,ti

#10 'stomach extirpation':ab,ti

#11 'stomach resection':ab,ti

#12 'stomach transection':ab,ti

#13 #5 OR #6 OR #7 OR #8 OR #9 OR #10 OR #11 OR #12

#14 'endoscopic surgery'/exp

#15 'endoscopic resection':ab,ti

#16 'resection, endoscopic':ab,ti

#17 'surgery, endoscopic':ab,ti

#18 'surgery, video assisted':ab,ti

#19 'surgery, endoscopic':ab,ti

#20 'video-assisted surgery':ab,ti

#21 'surgical procedures, endoscopic':ab,ti

#22 'endoscopic submucosal dissection'/exp

#23 'esd (endoscopic submucosal dissection)':ab,ti

#24 'endoscopic mucosal resection'/exp

#25 'emr (endoscopic mucosal resection)':ab,ti

#26 'endoscopic aspiration mucosectomy':ab,ti

#27 'endoscopic mucosa resection':ab,ti

#28 'endoscopic mucosectomy':ab,ti

#29 #14 OR #15 OR #16 OR #17 OR #18 OR #19 OR #20 OR #21 OR #22 OR #23 OR #24 OR #25 OR #26 OR #27 OR #28

#30 #4 AND #13 AND #29
